# The Study of Dynamic Characteristic of Acupoints Based on the Primary Dysmenorrhea Patients with the Tenderness Reflection on *Diji* (SP 8)

**DOI:** 10.1155/2015/158012

**Published:** 2015-06-16

**Authors:** Sheng Chen, Yanhuan Miao, Yinan Nan, Yanping Wang, Qi Zhao, Enhui He, Yini Sun, Jiping Zhao

**Affiliations:** ^1^Acupuncture and Moxibustion Department, Dongzhimen Hospital, Beijing University of Chinese Medicine, No. 5 Haiyuncang, Dongcheng District, Beijing 100010, China; ^2^College of Acupuncture-Moxibustion and Tuina, Beijing University of Chinese Medicine, No. 13 of the North 3rd East Road, Chaoyang District, Beijing 100029, China; ^3^International Medical Center, China-Japan Friendship Hospital, No. 2 Yinghua East Street, Chaoyang District, Beijing 100029, China

## Abstract

In TCM theory, acupoint is not a fixed point but a portal with dynamic characteristics where the channel *qi* enters and flows out. The dynamic characteristics have been verified primarily by detecting the tenderness reaction on *Diji* (SP 8) in primary dysmenorrhea patients. In this study, finger pressing and algometer were applied in *Diji* (SP 8) area of participants in menstrual period and nonmenstrual period, respectively, to detect the tenderness occurrence rate, the VAS score of the tenderest point, the tenderness threshold of the tenderest point, and the location of the tenderest point. The result suggests that the acupoint may not be a fixed location but a point in a dynamic state within a certain range in time and space varying with different physiological and pathological status.

## 1. Background

Acupoints are specific locations where* qi* and blood of meridians and* Zang-fu* organs infuse and also where diseases are reflected and the acupuncture needles are applied [1]. Therefore, acupoint is the essential factor in acupuncture diagnosis and treatment. Each acupoint has its own name, location, specificity, function, and so forth, while location is considered the most essential and fundamental. Without accurate and precise location, it is likely neither to examine the reaction of the acupoint in pathological condition, nor to find the point with therapeutic effects. Because of this, the standardization of the acupoint location is considered as the key in the history of Chinese acupuncture and moxibustion standardization. Since the official promulgation of the* location of acupoints* (national standard) in 1990, updated standards of the acupoint locations continued to be introduced one after the other in the years 2006 and 2010. The standardization of the acupoint location has to some extent normalized clinical operation and promoted the dissemination and development of the acupuncture and moxibustion worldwide. However what catches our attention is that while promoting the standard of the acupoint location, parts of the clinical practitioners stick too much to the fixed location of the acupoint and ignore the importance and necessity of searching and seeking acupoint along the meridian, leading to dissatisfactory clinical effect.

According to the description in the* Yellow Emperor's Inner Canon, the classic of Traditional Chinese Medicine*, acupoint is a portal where the channel* qi* enters and flows out and not a fixed point attaching to the skin, vessel, muscle, tendon, and bone. Therefore, acupoint is not isolated or static structure in the body. It is related to the movement of channel* qi*. It is not only able to reflect the changes of* qi* in channel, but also able to be used for adjusting the channel* qi*, showing dynamic characteristics. Whereas most of the researches on dynamic performance of acupoints have been theoretical, clinical research is lacking.

*Diji* (SP 8) is the most important and commonly used point for the treatment of dysmenorrhea. According to the theory of traditional Chinese medicine (TCM),* Diji* (SP 8) is the* Xi*-cleft point of the Spleen meridian of Foot* Taiyin*, where the meridian* qi* accumulates deeply and is suitable for treating acute pain and blood disease. Primary dysmenorrhea, a medical condition of cramping pain in the lower abdomen occurring before and during menstruation, is just ascribed to acute pain and blood disease in TCM. Therefore, this research focuses on patients with primary dysmenorrhea to observe the changes in tenderness in* Diji* (SP 8) in different physiological and pathological states, so as to explore the dynamic characteristic of acupoint and to provide clinical data for the study of acupoint dynamism from the clinical perspective.

## 2. Materials and Methods

### 2.1. Setting and Participants

30 patients with primary dysmenorrhea were recruited as the observation group between April and December of 2013 in* Dongzhimen* Hospital affiliated to Beijing University of Chinese Medicine. 30 healthy female volunteers from the Beijing University of Chinese Medicine were recruited as the control group during the same period of time.

As some patients with primary dysmenorrhea may resolve or be relieved spontaneously after giving birth, all the participants included were nulliparous, so as to reduce selection bias. In the observation group, the oldest participant was 34 years old, the youngest was 21 years old, and the mean of their age was 26 ± 5 years. In the control group, the oldest was 32 years old, the youngest was 32 years old, the smallest age was 23 years, and the mean of their age was 25 ± 2 years. After the statistical analysis of the distribution of age and disease duration between the control group and the observation group, the differences were not statistically significant. See details in Table 1.

### 2.2. Diagnostic Criteria, Inclusion Criteria, and Exclusion Criteria

#### 2.2.1. Observation Group

*(1) Diagnosis Criteria*. Referring to the Canadian Department of Gynecology and Obstetrics Association in 2005* primary dysmenorrhea clinical guideline* [2], standards are as follows.

(1) The first one is women with lower abdominal pain that begins somewhere between several hours before and a few hours after the onset of the menstrual bleeding, usually persisting up to 2-3 days; (2) the pain is characteristically colicky or dull and located in the midline of the lower abdomen but may extend to both lower quadrants, the lumbar area, and the thighs; (3) the pain is frequently associated with symptoms including diarrhea, nausea, vomiting, fatigue, light-headedness, headache, dizziness, and, rarely, syncope and fever; (4) the symptoms are more or less reproducible from one menstrual period to the other; (5) type B ultrasonic examination and gynecological examination exclude the organic pathological changes in the reproductive organs.

*(2) Inclusion Criteria*. These criteria include the following: (1) patients who fulfill the diagnostic standard of the primary dysmenorrhea; (2) patients of ages between 18 and 35 years; (3) patients who have never given birth; (4) patients who have disease duration ≥6 months; (5) patients who have regular menstrual cycle (28 ± 7) d; (6) patients who have abdominal pain which occurs 48 hrs within the onset of menstruation; (7) patients with COX Dysmenorrhea Symptom Scale (CMSS) [3] total score ≥8; (8) patients with VAS score of the abdomen pain ≥40 during the attack of dysmenorrhea; (9) patients who have no participation in any other medication or modality clinical trials; (10) patients who signed informed consent.

*(3) Exclusion Criteria*. These criteria include the following: (1) patients with life threatening disorders, such as cardiovascular, liver, kidney, hematopoietic system disorders, and mental diseases; (2) patients who have skin problem on and near* Diji* (SP 8), such as soft tissue damage, ulceration, scar, and skin calluses; (3) patients who received other related treatments within a month or intake of pain killers, sedatives, and hormone drugs within 2 weeks; (4) patients who are physically weak or judged not suitable to participate in this research by researchers.

#### 2.2.2. Control Group

*(1) Inclusion Criteria*. These criteria include the following: (1) healthy women with no abnormalities in the physical examination within the recent semester; (2) patients of ages between 18 and 35 years; (3) patients who have never given birth; (4) patients who have no history of dysmenorrhea in the past; (5) patients who have mostly regular menstrual cycle (28 ± 7) d; (6) patients who signed informed consent.

*(2) Exclusion Criteria*. These criteria include the following: (1) patients suffering from frequent lower abdominal pain of unknown reason; (2) patients with skin problems on and near* Diji* (SP 8), such as soft tissue damage, ulceration, scar, and skin calluses; (3) patients who received other related treatments within a month or intake of pain killers, sedatives, and hormone drugs within 2 weeks; (4) patients with mental disorders or judged not suitable to participate in this research by researchers.

### 2.3. Tenderness Detection on* Diji* (SP 8)

In order to guarantee the quality of the study, every segment of the operation was performed by the same researcher, who had received training over six months, to ensure the standardization and unity of the operation.

#### 2.3.1. Detecting Point

*Diji* (SP 8) on both sides of the legs and their surrounding areas of a total of 60 participants in both the observation group and control group was detected.

The participants were instructed to lie supine with legs straightened in a relaxed manner while fully exposing the parts below the knees.

**Figure 1:**
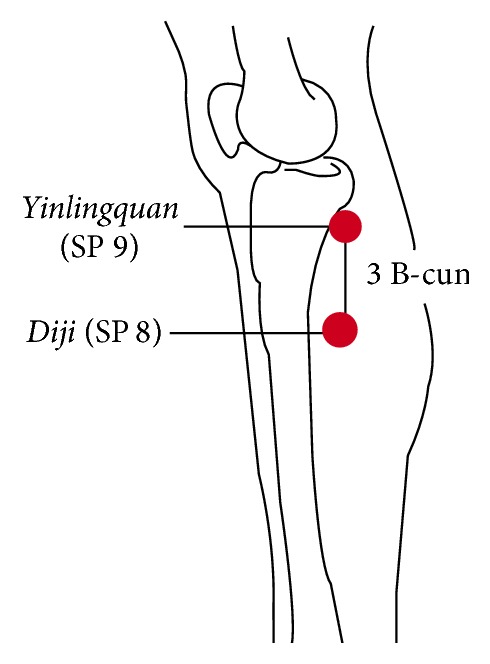
Standard position of* Diji* (SP 8).

*Diji* (SP 8) was located referring to* WHO standard acupuncture point locations in the Western Pacific Region* [4], which is “on the tibial aspect of the leg, posterior to the medial border of the tibia, 3 B-cun inferior to SP9” (Figure 1). Mark this point as the standard position of* Diji* (SP 8).

#### 2.3.2. Detecting Tenderness with VAS [5]

Starting from the standard position of* Diji* (SP 8), the researcher pressed spirally with the tip of the thumb pulp in a circular area 2 cm long in radius. The intensity of the pressing force was consistent and even to the level of muscle. The tenderness was recognized when pain, soreness, or distension sensation was expressed through the immediate and fleeting reactions of participants' eyes or words.

When tenderness reaction appeared, the participants were instructed to face the reverse side of the VAS card without graduation and then move the cursor to the position that best represented the pain intensity. The researcher facing the side with calibration recorded VAS scores and marked the position. The point with highest VAS scores was the tenderest point; if there is no tenderness upon pressure, the result was just recorded with no VAS detection.

#### 2.3.3. Detecting Pain Threshold of the Tenderest Point

**Figure 2:**
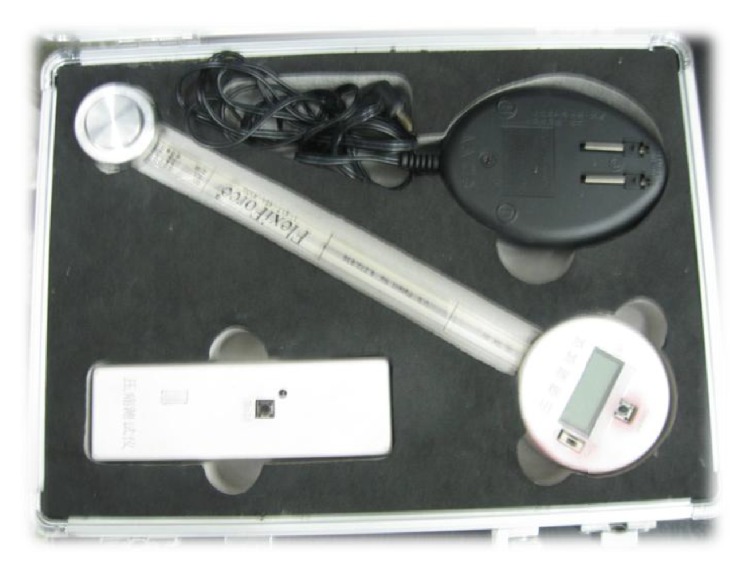
Algometer.

After the VAS assessment, the participants were instructed to rest for 10 minutes. Then, the pain threshold of the tenderest point was detected using an algometer (National Patent number: ZL200520142236.5; Product Publication number: CN2862954; Manufacturer: Institute of Orthopedics and Traumatology Affiliated to Chinese Academy of Traditional Chinese Medicine Science; Place of Production: Beijing) (Figure 2).

Firstly set the tester to zero. Then put the probe tip (0.5 cm in diameter) of the tester vertically onto the mark point. Apply pressure gradually and evenly downward (the maximum pressure should not exceed 600 kpa for avoiding tissue damage caused by excessive force). Once the participant reports pain or a radiating pain was elicited, then the algometer was removed and the data on the tester screen was recorded as the pain threshold value. Such a procedure was conducted on both sides of the* Diji* (SP 8) area, with the left one coming first.

#### 2.3.4. Measure the Location of the Tenderest Point

The distance between the center of the tenderest point and the standard position of* Diji* (SP 8) was measured with a soft tape measure and then recorded.

#### 2.3.5. Detecting Time Point

1. For the observation group, detection of time point occurred during the first day or second day following the onset of dysmenorrhea and the seventh day after menstruation (nonmenstrual period).
2. For the control group, detection of time point took place the first or second day following menstrual onset and the seventh day after menstruation (nonmenstrual period).

### 2.4. Statistical Analysis

SPSS17.0 statistical software was used for analysis. Count data were tested using *x* ^2^ test. One-way ANOVA was adopted for sets of normally distributed data which went through paired comparison using S-L-D method. *t*-test was used for the data from two groups and the data were expressed by the mean plus or minus standard deviation. Skewed data were tested using the nonparametric Wilcoxon test and expressed in M (QR), that is, the median (interquartile range). All statistical tests were tested and verified using the two-sided test. *P* ≤ 0.05 was considered statistically significant.

## 3. Results

### 3.1. Comparisons of Tenderness Occurrence Rate in* Diji* (SP 8) Area

In observation group, there were 5 one-side tenderness cases and 1 pain-free case; the rest of the cases presented with tenderness on both sides in* Diji* (SP 8) area during acute onset period. Total TOR was 88.3%. During nonmenstrual period, there were 8 one-side tenderness cases and 7 pain-free cases, and the rest presented with tenderness on both sides. Total TOR was 63.3%. In control group, there were 11 one-side tenderness cases and 9 pain-free cases, and the rest of the cases presented with tenderness on both sides in* Diji* (SP 8) area during menstrual period. Total TOR was 51.7%. During nonmenstrual period, there were 5 one-side tenderness cases and 18 pain-free cases, and the rest presented with tenderness on both sides. Total TOR was 31.7%.

**Figure 3:**
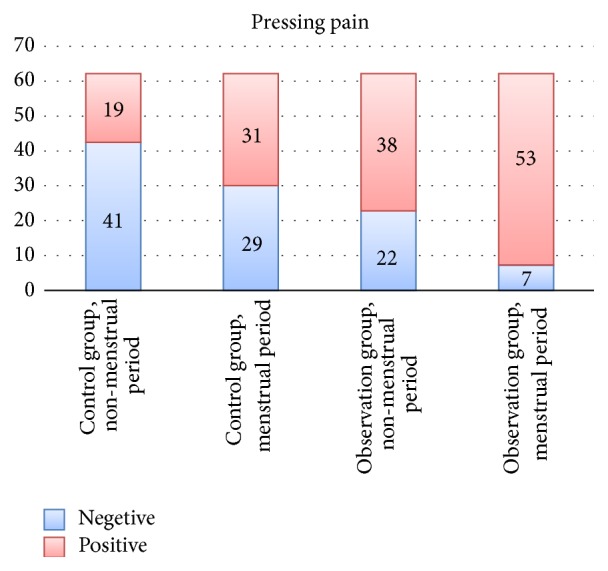
Comparison of the TOR of each group during menstrual and nonmenstrual period.

We used Chi-squared test to compare the TOR between two groups and two menstrual periods (corrected value *P*′ = 0.00833). In the observation group, the TOR during menstrual period was significantly higher than that in the nonmenstrual period, *P* < *P*′. In the control group, the TOR during menstrual period was also significantly higher than that in the nonmenstrual period, *P* < *P*′. During nonmenstrual period, the TOR of the observation group was also significantly higher than that of the control group, *P* < *P*′ (Figure 3, Table 2).

### 3.2. Comparison of VAS Score of the Tenderest Point in* Diji* (SP 8) Area

**Figure 4:**
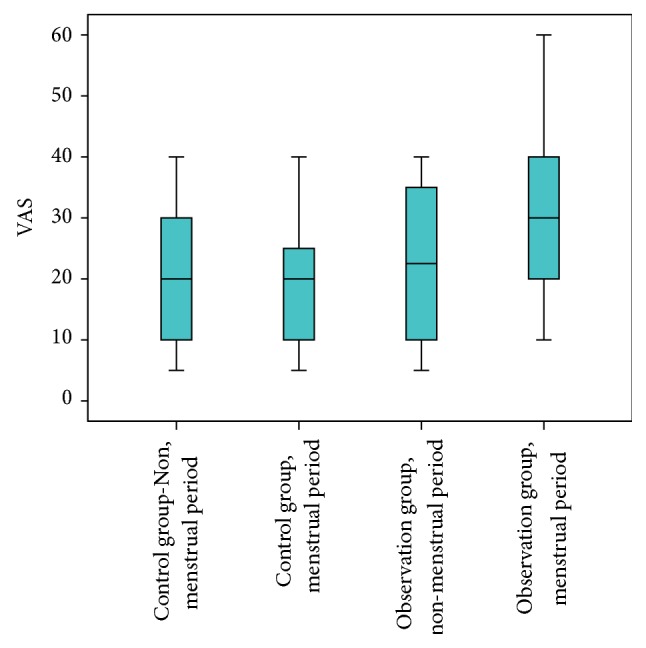
Comparison of VAS score between menstrual period and nonmenstrual period in two groups (mm).

Using nonparametric test, we compared VAS score between groups in menstrual period and nonmenstrual period (corrected value *P*′ = 0.00833). VAS score of the observation group in the menstrual period was significantly higher than that in the nonmenstrual period, while there was no significant difference between two periods in the control group. In menstrual period, VAS score of the observation group was significantly higher than that of the control group, *P* < *P*′. In nonmenstrual period, VAS score of the observation group was higher than that of the control group, while there was no significant difference, *P* > *P*′ (Figure 4, Table 3).

### 3.3. Comparison of Tenderness Threshold Value of the Tenderest Point in* Diji* (SP 8) Area

**Figure 5:**
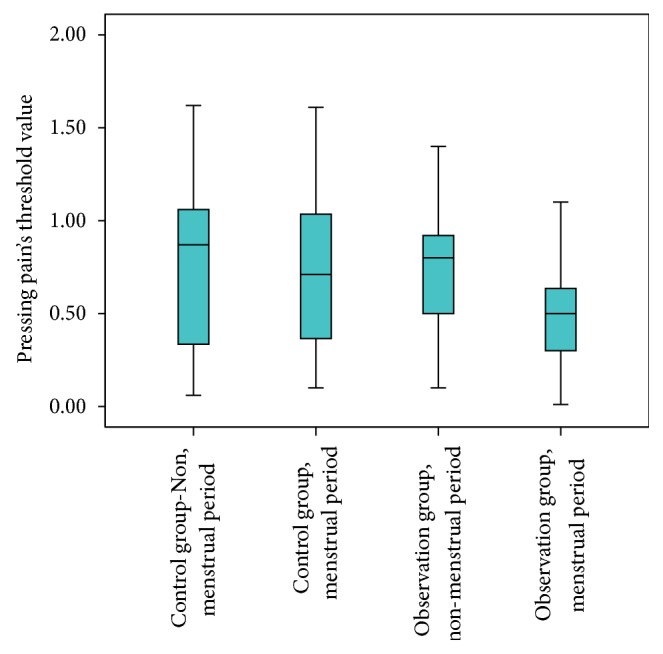
Tenderness threshold value in* Diji* (SP 8) area in menstrual and nonmenstrual period (kPa).

We used one-way ANOVA to analyze two groups' tenderness threshold value of the tenderest point in* Diji* (SP 8) area in menstrual and nonmenstrual period. The results showed *F* = 4.983, *P* = 0.003 < 0.05, indicating that there was difference in threshold value between two groups in menstrual or nonmenstrual period. S-L-D method was used for further analysis. Tenderness threshold value in the menstrual period of the observation group was significantly lower than that in the nonmenstrual period, *P* < *P*′, while there was no significant difference between two periods in the control group. In menstrual period, tenderness threshold value of the observation group was significantly lower than that of the control group, *P* < *P*′. In nonmenstrual period, tenderness threshold value of the observation group was lower than that of the control group, while there was no significant difference, *P* > *P*′ (Figure 5, Table 4).

### 3.4. Location of the Tenderest Point in* Diji* (SP 8) Area

**Figure 6:**
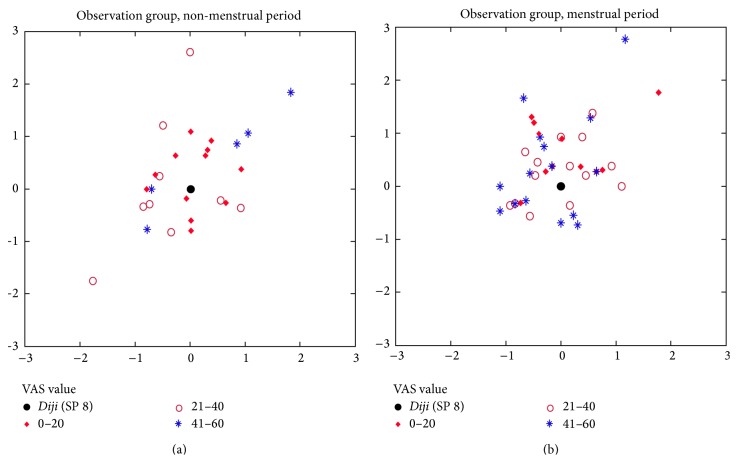
Location of the tenderest point in* Diji* (SP 8) area (cm).

Our results showed overlaps between the tenderest point and standard point in observation group during menstrual period and nonmenstrual period. Overlap rate was 22.6% and 28.9%, respectively. We measured the distance between the tenderest point and the standard point and came up with the following conclusion: if we set standard point of* Diji* (SP 8) as datum point, distribution range of the tenderest point in observation group was 0.565–0.903 cm in menstrual period and 0.515–0.974 cm in nonmenstrual period, respectively (Figure 6, Table 5).

## 4. Discussions

The dynamic characteristic of the acupuncture point is one of the hot topics in the acupuncture research. Previous study has shown that acupuncture points are of dimensional structure located in the interstice within the skin, vessel, muscle, sinew, bone, and even viscera, rather than fixed points. Their location may be influenced by several factors such as different physiological changes and pathological conditions of the* Zang-fu* organs and channels and the external environment and individual variety. Therefore, acupuncture points possess the individualized and dynamic characteristic [6].

There are four reasons for applying the pressing examination on* Diji* (SP 8) of the patients who suffered from primary dysmenorrheal to study the dynamism of acupuncture points on reflecting disease in this research. First, according to the channel and acupuncture point theory in TCM,* Diji* (SP 8) is the* Xi*-cleft point of the Spleen meridian of Foot* Taiyin*.* Xi*-cleft points are where the meridian* qi* accumulates deeply and are indicated for the acute and pain disease of the respective* Zang-fu* organs and meridians, while the* Xi*-cleft points of the Yin meridians are also indicated for blood diseases [7]. Primary dysmenorrhea, a medical condition of cramping pain in the lower abdomen occurring just before and during menstruation, is ascribed to acute pain and blood disease in TCM. Therefore,* Diji* (SP 8) is the most important and commonly used point for the treatment of dysmenorrhea [8–11]. Second, the acupuncture points relate closely to the internal* Zang-fu* organs through the pathway of the meridians; thereby the condition of the diseased* Zang-fu* organs will be reflected on the acupuncture point through the transmission of the meridians [12]. Third, though there are various examination methods and techniques for the reflection effect of acupuncture point, such as detecting the electric currency and electrical resistance of the point [13–17] and the infrared thermal imaging technique [18, 19], the most commonly used, convenient, and consensus method is detecting the tenderness and pain threshold [20–22]. Fourth, there are few researches and reports on reflecting effect of* Diji* (SP 8) on the dysmenorrhea.

*Diji* (SP 8) of the 30 patients with primary dysmenorrhea and 30 healthy women was palpated by hand and detected by algometer. The result of the research and the concerning issues are discussed as follows.

1. The tenderness occurrence rates and the VAS score of* Diji* (SP 8) in menstrual period of the observation group were higher than that in nonmenstrual period and the menstrual period in the control group. The tenderness threshold of* Diji* (SP 8) in the menstrual period of observation group was lower than that in the nonmenstrual period of observation group and the menstrual period in the control group. The result showed that there exist dynamic characteristics in* Diji* (SP 8) in the tender reaction in both the physiological and pathological conditions, including the different stages in the pathological condition. The tenderness reaction was more likely to occur and more intensive and sensitive in the menstrual period of the primary dysmenorrhea patient.
2. There is no statistical difference in the tenderness occurrence rates, VAS score, and tenderness threshold in both the menstrual period and nonmenstrual period in the control group. It indicates that there is no remarkable change in the tenderness reaction on* Diji* (SP 8) in the physiological state and the alteration of the physiological rhythm. Comparing the data in the nonmenstrual period of the test and control group, the tenderness occurrence rate of observation group is higher than that of the control group, but there is no statistical difference between the two groups in VAS value and the tenderness threshold. The results might be considered as follows: in the nonmenstrual period, the dysmenorrhea patients were still in the pathological states of blood deficiency failing to nourish the uterus or blood stasis blocking the meridian in the uterine, which makes* Diji* (SP 8) become more sensitive to pressing; however the severity was not intensive. Or it might be that the patients enrolled were not enough to show the statistical difference. From an anatomical perspective, there are parts of the saphenous nerve in shallow layer of* Diji* (SP 8) area and sympathetic nerve governing the myometrium contraction coming from the same nerve segments. Accordingly, the same situation also occurs between the tibial nerve in deep layer and parasympathetic nerve controlling the sense of uterus. It can thus be seen that* Diji* (SP 8) has a close relationship with uterus in anatomy [23]. However, the results still possibly indicate that* Diji* (SP 8) might reflect the pathological condition in the menstrual period of the dysmenorrhea patients, which also validate the viewpoint that pathological reaction in meridian and acupuncture points relates to timing [24].
3. This research found out that, in* Diji* (SP 8) area, the tenderest points were divergent from the standardized location of* Diji* (SP 8) in the majority of dysmenorrheal patients, which indicates that the location of* Diji* (SP 8) in pathological state is different from the standardized location. Acupuncture point is both the reflective point of diseases and where the needles and moxibustion are applied for the treatment of disease in the meridian and acupuncture point theory [25]. Wang illuminates the process acupuncture reflecting disease. Acupuncture point is where both the pathological factors and meridian* qi* exist when patient suffered from a disease. If the meridian* qi* fails to dispel the pathological* qi* out of the body in time, the pathological factors accumulate in the acupuncture point; consequently, there might be pathological reactions such as pain, tenderness, and other changes on the acupuncture point [26]. Zeng et al. put forward the viewpoint that, in the pathological state, the surface reflection area of acupuncture varies with the condition of the disease; it increases when disease gets worse, decreases when disease gets better, and disappears when disease is cured [27, 28]. As to the enlargement of the surface areas around acupoint reflecting diseases, Yu et al. believe that it relates to the facilitation and sensitization of the spinal cord caused by visceral disorders, where the information coming from the body surface and viscera are assembled [29]. According to the theory of “painful locality taken as an acupoint,” the tenderest point in* Diji* (SP 8) area might be the veracious point of* Diji* (SP 8) in the pathological state and it might also be the most effective point for treatment. We have found in clinic that, in the acute stage of dysmenorrhea, the part between* Sanyinjiao* (SP 6) and* Yinlingquan* (SP 9) on the pathway of Spleen meridian is the main reaction region. Tender, sore, or distending points, especially distinctive around the* Sanyinjiao* (SP 6),* Diji* (SP 8), and* Yinlingquan* (SP 9) area, will be found when palpating along the meridian. Needling on the tender point will bring instinctive effect of relieving the pain. Of course, large scale clinical trial is required to confirm our clinical observation.
4. There have been 2 Chinese national standards of acupuncture location (1990 and 2006) and* Standard Acupuncture Nomenclature* (second edition) was published in 1993 [30–34]. The standardization of acupuncture point location helps to standardize the needle manipulation and also promote the spreading of acupuncture worldwide. In standardized acupuncture point location, a vertical and horizontal coordinate method is adopted as much as possible to locate the acupuncture point; for example,* Zusanli* (ST 36) is “on the anterior aspect of the leg, on the line connecting ST35 with ST41, 3 B-cun (proportional bone cun) inferior to ST35.” Then,* Zusanli* (ST 36) is stated as the crossing point of the two intersecting lines. Accordingly, this locating method makes some acupuncture practitioners stick to the standardized location of acupoints and ignore the dynamic character of the acupoint. However, this might result in the less satisfied effect in clinic. The description of the location of some extra acupuncture point in textbook of acupuncture and moxibustion before the standardization of acupuncture point location in China embodies the dynamic character of acupuncture point. For example,* Lanwei* (appendix) point is located around 2 cun inferior to* Zusanli* (ST 36), and* Danang* (gallbladder) is 1-2 cun inferior to* Yanglingquan* (GB 34) [35].
5. In acupuncture clinical trials, selecting the nontraditional-acupoint site near the acupoint for treatment as the placebo control is called adjacent nonacupoint control method [36], which is the main method in the nonacupoint controlled trials [37]. The way of selecting the nonacupoint is reasonable or does not determine the validity of the control and the authenticity and veracity of the results. This research as well as others [38–41] all revealed that the location and size of the acupoints may change with the different conditions of the body, and in the pathological condition the size on the body surface may even get large, which embodies the dynamic characteristic of the acupuncture points. Therefore, the adjacent nonacupoint might just be the site of acupuncture point for treatment, and needling the adjacent nonacupoint, possibly, might produce the same or even better effect compared to that of the acupuncture point in the standardized location. Thereby, the result of clinical trials adopting the nearby nonacupoint as a control is debatable.

This research is an exploratory study on the dynamism of acupuncture points through detecting the tenderness reaction on* Diji* (SP 8) in primary dysmenorrhea patients. Further studies are required for answering questions concerning the dynamism of acupuncture points, such as what the changes of the acupuncture points in different physiological and pathological conditions as well as in different stages of the pathological conditions are and whether or not needling on the tender site of the acupuncture point might improve the treatment effect.

## Figures and Tables

**Table 1:** Comparison ages between observation group and control group (unit: age).

Group	Number of cases	Age		
		Smallest value	Largest value	M (QR)
Observation group	30	21	34	26 (5)
Control group	30	23	32	25 (2)
*Z*				−0.88
*P*				0.378

**Table 2:** Comparison of the TOR in *Diji* (SP 8) area between menstrual and nonmenstrual period.

Group			Menstrual period	Nonmenstrual period	*x* ^2^	*P*
Observation group	Tenderness	Positive	53	38		
		Negative	7	22	10.231	0.001
	TOR		88.3%	63.3%^*^		
Control group	Tenderness	Positive	31	19		
		Negative	29	41	4.937	0.026
	TOR		51.7%^*^	31.7%^#^		
*x* ^2^			19.206	12.063		
*P*			0.000	0.001		

Note: corrected value *P*′ = 0.00833. ^*^Compared with observation group in menstrual period, *P* < *P*′. ^#^Compared with observation group in nonmenstrual period, *P* < *P*′.

**Table 3:** Comparison of VAS score between menstrual and nonmenstrual period in two groups (mm).

Group		Menstrual period	Nonmenstrual period	*Z*	*P*
Observation group	Number of effective values	53	38	−2.646	0.0081
	Minimum value	10	5		
	Maximum value	60	40		
	M (QR)	30 (20)	22.5 (25)^*^		
	Mean rank	88.76	66.12		
Control group	Number of effective values	31	19	−0.182	0.856
	Minimum value	5	5		
	Maximum value	40	40		
	M (QR)	20 (15)^*^	20 (20)		
	Mean rank	54.89	57.50		
*Z*		−3.705	−0.780		
*P*		0.000	0.436		

Note: corrected value *P*′ = 0.00833. ^*^Compared with observation group in menstrual period, *P* < *P*′.

**Table 4:** Tenderness threshold value in *Diji* (SP 8) area in menstrual and nonmenstrual period (kPa).

Group		Menstrual period	Nonmenstrual period	Mean difference	*P*
Observation group	Number of effective values	53	38	−0.251	0.001
	Minimum value	0.01	0.1		
	Maximum value	1.2	1.6		
	$\overset{-}{x}\pm s$	0.497 ± 0.040	0.748 ± 0.375^*^		
Control group	Number of effective values	31	19	−0.027	0.801
	Minimum value	0.1	0.06		
	Maximum value	1.61	1.62		
	$\overset{-}{x}\pm s$	0.724 ± 0.385^*^	0.751 ± 0.468		
Mean difference		0.227	−0.003		
*P*		0.006	0.979		

Note: corrected value *P*′ = 0.00833. ^*^Compared with observation group in menstrual period, *P* < *P*′.

**Table 5:** Distance between the tenderest point and standard point in *Diji* (SP 8) area (cm).

		Number of effective values	Overlap rate with standard point	Minimum value	Maximum value	M (QR)	95% CI
Observation group	Menstrual period	53	22.6%	0	3	0.7 0.6	0.565–0.903
	Nonmenstrual period	38	28.9%	0	2.6	0.7 (1)	0.515–0.974
